# Coronavirus Disease 2019 (COVID-19): A Systematic Review of Pregnancy and the Possibility of Vertical Transmission

**Published:** 2020

**Authors:** Mohammad Ali Ashraf, Pedram Keshavarz, Parisa Hosseinpour, Amirhossein Erfani, Amirhossein Roshanshad, Alieh Pourdast, Peyman Nowrouzi-Sohrabi, Shahla Chaichian, Tahereh Poordast

**Affiliations:** 1- Student Research Committee, Shiraz University of Medical Sciences, Shiraz, Iran; 2- Department of Radiology, Medical Imaging Research Center, Shiraz University of Medical Sciences, Shiraz, Iran; 3- School of Medicine, Islamic Azad University, Kazeroun branch, Kazeroun, Iran; 4- Thoracic and Vascular Surgery Research Center, Shiraz University of Medical Sciences, Shiraz, Iran; 5- Department of infectious diseases, Tehran University of Medical Sciences, Tehran, Iran; 6- Iranian Research Center for HIV/AIDS (IRCHA), Tehran, Iran; 7- Department of Biochemistry, Student Research Committee, Shiraz University of Medical Sciences, Shiraz, Iran; 8- Pars Advanced and Minimally Invasive Medical Manners Research Center, Pars Hospital, Iran University of Medical Sciences, Tehran, Iran; 9- Department of Obstetrics and Gynecology, School of Medicine, Shiraz University of Medical Sciences, Shiraz, Iran

**Keywords:** Coronavirus Disease 2019 (COVID-19), Neonatal outcomes, Pregnancy, Pregnant women, SARS-CoV-2, Systematic review, Vertical transmission

## Abstract

**Background:**

There is a growing need for information regarding maternal and neonatal outcomes during coronavirus pandemic. In this study, a comprehensive investigation was done regarding the possibility of vertical transmission using the available data in the literature.

**Methods:**

A systematic search was conducted using electronic databases, including PubMed, Scopus, Web of Science, Embase, and Scholar. All studies containing infected COVID-19 pregnant women who had given birth were included, and the search was done up to April 14, 2020.

**Results:**

Overall, 21 articles were reviewed, and clinical characteristics of 90 pregnant patients and 92 neonates born to mothers infected with COVID-19 were reviewed. The most common symptoms included fever, cough, and dyspnea. The main laboratory findings included leukocytosis, lymphopenia, thrombocytopenia, and elevated C-reactive protein. The most commonly reported complications were preterm labor and fetal distress. Three mothers were admitted to ICU and required mechanical ventilation; among them, one died, and one was on extracorporeal membrane oxygenation. Overall, 86 neonates were tested for the possibility of vertical transmission and 82 cases were negative in RT-PCR, while 4 were positive. Out of 92 neonates, one died, and one was born dead. Nineteen patients reported having no symptoms, while breathing problems and pneumonia were reported as the most common neonatal complications.

**Conclusion:**

There were no differences in the clinical characteristics of pregnant women and non-pregnant COVID-19 patients. COVID-19 infection has caused higher incidence of fetal distress and premature labor in pregnant women. Although the possibility of vertical transmission in infected pregnant women is rare, four neonates’ test results for COVID-19 infection were positive in this review.

## Introduction

Since the onset of coronavirus disease 2019 (COVID-19) in Wuhan (1), it has infected more than 7.1 million people around the world. COVID-19 is now considered a pandemic since World Health Organization (WHO) announcement on March 11, 2020 (2). Pregnant women are one of the endangered groups who need special attention in this situation.

Women’s physiological modulation in the immune system during pregnancy puts them and their neonates at increased risk of negative outcomes of COVID-19 infection (3).

Various viral infections such as cytomegalovirus, severe acute respiratory syndrome (SARS), and Middle-East respiratory syndrome (MERS) during pregnancy can be associated with a higher incidence of preterm labor, congenital fetal anomalies of the central nervous system and the cardiovascular system, and chorioamnionitis. Besides, transplacental passage of the virus and the following infection has adverse effects on the fetus (4). As seen in the severe acute respiratory syndrome epidemic in 2004, severe acute respiratory syndrome-related coronavirus (SARS-CoV) was associated with higher rates of miscarriage, preterm delivery, and intrauterine growth restriction (5). However, there is no conclusive opinion in this regard.

According to Centers for Disease Control and Prevention (CDC), much is still unknown about the effects of COVID-19 on the pregnancy and neonatal outcome (6). Therefore, it is important to gain knowledge about pregnancy outcomes during the COVID-19 pandemic, including the possibility of vertical transmission, the severity of symptoms in pregnant women, potential complications during pregnancy, and the condition of newborns of an infected mother. However, there is no comprehensive information regarding its effects on pregnancy.

In this article, an attempt was made to conduct a systematic literature review to demonstrate the effects of COVID-19 on pregnant women and the possibility of vertical transmission.

## Methods

### Study design and search strategy:

A systematic search was conducted using the electronic databases, including PubMed, Scopus, Web of Science, Embase, and Scholar up to April 14, 2020. The Preferred Reporting Items for Systematic Reviews and Meta-analyses (PRISMA) was used for the selection process in our systematic review (7). The initial search of articles was done using the following search pattern: [key terms for vertical transmission] AND [key terms for COVID-19]. The keywords details and full search strategy used in PubMed are provided in the supplementary appendix. This study was registered at Shiraz University of Medical Sciences (SUMS) ethics committee (IR.SUMS.REC.1399.172).

### Study selection:

Reported studies consisted of case reports, case series, and letters containing case information. There was no cohort study or randomized clinical trials in the final screened articles. Studies that met the following criteria were included in this research:

1. Studies that were reporting pregnant women with confirmed COVID-19 who had recently given birth.
2. The newborns of these mothers were subjected to test for COVID-19.
3. The included studies must at least contain the results of both mothers and newborns samples for COVID-19 test. Only studies that present the outcome of vertical transmission were used in this review.

Duplicate studies, abstracts, conferences, review articles, non-original articles, non-English language studies, and studies that did not contain results for COVID-19 tests of mothers and newborns were excluded from this systematic review. Also, studies containing pregnant women whose COVID-19 infections were not confirmed or those who had not given birth were excluded.

### Data extraction and quality assessment:

A predefined data extraction list was designed by the authors. Therefore, a data extraction list was used to extract the required data from the included studies. The extraction list included the following:

First author’s name, publication date, study design, sample size, and medical details of mothers and their newborns. The medical details used in the analysis consisted of demographic, clinical, para-clinical data, and the outcomes of mothers and their newborns. The discrepancies in the data and the completed data sheets were reviewed by another author. Quality assessment was done using the tool for evaluating the methodological quality of case reports and case series proposed by Murad et al. (8). This tool evaluates the quality of the studies with 8 questions categorized in 4 different domains: the selection of the cases (1 question), the ascertainment (2 questions) and the causality (4 questions) of the exposure and outcome, and the reporting of the cases (1 question). However, responding to 2 questions in the causality domain was not possible in this study. Therefore, the quality of all the articles were independently assessed by two authors, and disagreements were discussed with the third author and resolved.

### Outcomes:

The clinical characteristics of COVID-19 in pregnancy, the risk of pregnancy complications, mortality of mothers and their newborns, and the possibility of vertical transmission were reported in this systematic review.

## Results

**Figure 1.**
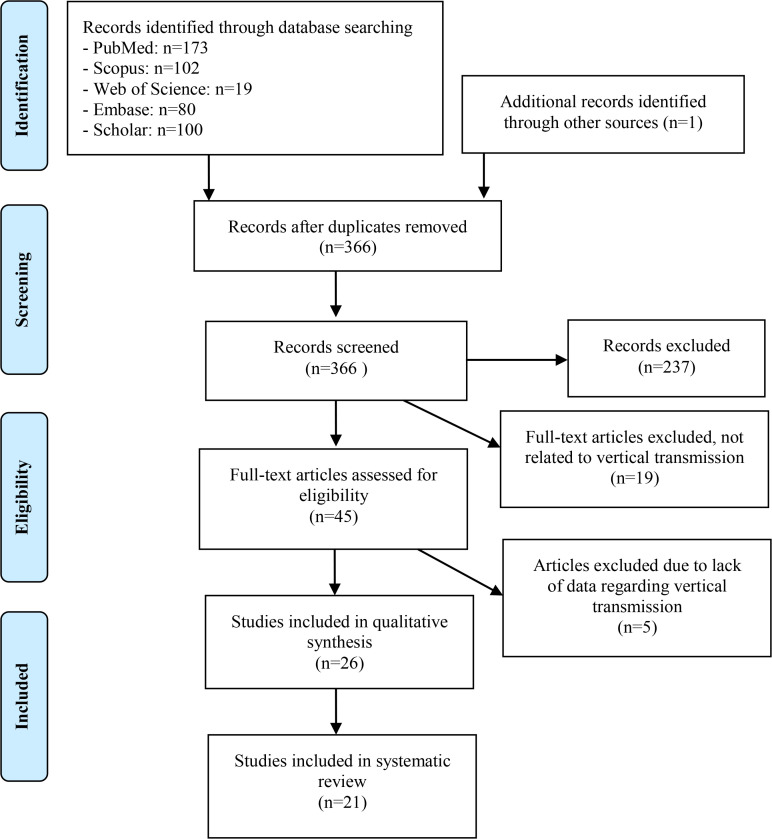
Flow diagram of study selection process

The systematic search resulted in 366 articles; 129 abstracts were screened. Full texts of 45 articles were reviewed, and 24 articles were excluded due to the lack of information regarding vertical transmission. All twenty-one remaining articles were included and reviewed in the study (9–29) (Figure 1).

Overall, 90 pregnant women were studied in which 84 were diagnosed with COVID-19 through positive reverse-transcriptase-polymerase-chain-reaction (RT-PCR) for SARS-CoV-2, and six were diagnosed based upon chest CT scan findings and history and physical exam. Furthermore, characteristics of 92 (Two sets of twins) neonates born to mothers infected with COVID-19 were also reviewed. Furthermore, the quality assessment table is provided in the supplementary appendix (Table 1A).

**Table 1A.** Quality assessment of all 21 included studies

**Study ID**	**Selection**	**Ascertainment**		**Causality**				**Reporting**

Quality assessment of the included case report and case series according to the four-domain tool proposed by Murad et al. ^*^								

	**Representativeness of the patients**	**Ascertained exposure**	**Ascertained outcome**	**Ruling out other causes**	**Challenge/rechallenge phenomenon**	**dose–response effect**	**Enough follow up time**	**Sufficient details of the cases**
**Chen (Huijun)**	No	Yes	Yes	Yes	N/A	N/A	Yes	Yes
**Chen (Rong)**	No	Yes	Yes	Yes	N/A	N/A	Yes	Yes
**Chen (Yan)**	No	Yes	Yes	Yes	N/A	N/A	Yes	Yes
**Dong**	No	Yes	Yes	Yes	N/A	N/A	Yes	Yes
**Gidlöf (Sebastian)**	No	Yes	Yes	Yes	N/A	N/A	Yes	Yes
**Huang**	No	Yes	Yes	Yes	N/A	N/A	No	No
**Iqbal**	No	Yes	Yes	Yes	N/A	N/A	Yes	Yes
**Kalafat**	No	Yes	Yes	Yes	N/A	N/A	No	Yes
**Khan (Suliman) #1**	No	Yes	Yes	No	N/A	N/A	Yes	Yes
**Khan (Suliman) #2**	No	Yes	Yes	Yes	N/A	N/A	Yes	Yes
**Lee**	No	yes	Yes	Yes	N/A	N/A	Yes	Yes
**Li (Yang)**	No	Yes	Yes	Yes	N/A	N/A	Yes	Yes
**Liao**	No	Yes	Yes	Yes	N/A	N/A	No	Yes
**Liu (Weiyong)**	Yes	Yes	Yes	Yes	N/A	N/A	Yes	Yes
**Liu (Yangli)**	Yes	Yes	Yes	No	N/A	N/A	Yes	Yes
**Wang (Shaoshuai)**	No	Yes	Yes	No	N/A	N/A	Yes	Yes
**Wang (Xiaotong)**	No	Yes	Yes	Yes	N/A	N/A	Yes	Yes
**Xiong**	No	Yes	Yes	Yes	N/A	N/A	No	Yes
**Zamaniyan**	No	Yes	Yes	Yes	N/A	N/A	Yes	Yes
**Zeng**	No	Yes	Yes	Yes	N/A	N/A	No	Yes
**Zhu**	No	Yes	Yes	Yes	N/A	N/A	Yes	Yes

* Murad MH, Sultan S, Haffar S, Bazerbachi F. Methodological quality and synthesis of case series and case reports. BMJ evidence-based medicine. 2018;23(2):60–3

### Clinical characteristics, diagnosis, and treatment

Based on the reviewed articles, the pregnant patients were aged between 22 to 40 years of age, who were between 28+2/7 to 40 weeks of gestational age (Third trimester). The most common symptoms included fever (N=47), cough (N=34), and dyspnea (N=12). The least were headache (N=3), nasal congestion (N=3), malaise (N=3), and chills (N=2). The reported temperatures were between 37 to 38.8°*C* (Low grade). Eleven patients reported fatigue, six patients reported myalgia, and five reported gastrointestinal symptoms such as diarrhea and nausea and vomiting was reported in one. Medical history taking revealed 37 patients to have a history of exposure to a patient infected with COVID-19 or high-risk environment such as hospitals for routine checkups. The time interval of onset of symptoms to delivery varied from the day of delivery to 37 days (Table 1).

**Table 1.** Maternal and neonates’ characteristics and outcomes among pregnant patients with COVID-19 infection

**Author/number of pregnant patients/infants**	**C/S or NVD**	**Mother’s RT-PCR results**	**Neonates RT-PCR results**	**Assessment of vertical transmission**	**Mother’s symptoms**	**Neonates symptoms after birth**	**Complications**	**Abnormal radiology finding in neonates**	**Mothers outcome**	**Neonates outcome**	**Preterm delivery**
Gidlöf et al. (9)/1/2	C/S	Positive	Negative	(2) Nasopharyngeal swab	Malaise, hoarseness, headache, fever	(1) Breathing problem first day, (1) Gastrointestinal reflux and cyanotic attack	None	NA	Alive	(2) Alive	(2) Positive
Huang et al. (10)/1/1	C/S	Positive	Negative	Nasopharyngeal swab	Dry cough	Not reported	Fetal distress	NA	Alive	Alive	Positive
Iqbal et al. (11)/1/1	NVD	Positive	Negative	Nasopharyngeal, amniotic fluid	Cough, fever, chills, myalgia	None	None	NA	Alive	Alive	Negative
Kalafat et al. (12)/1/1	C/S	Positive	Negative	Nasopharyngeal swab, cord blood, placenta	Dry cough, shortness of breath	None	None	NA	Alive (Intubated)	Alive	Positive
Lee et al. (13)/1/1	C/S	Positive	Negative	Nasopharyngeal swab, amniotic fluid, cord blood, placenta	Fever, sore throat, cough	None	None	NA	Alive	Alive	Negative
Liao et al. (14)/1/1	C/S	Positive	Negative	Nasopharyngeal swab, amniotic fluid, cord blood, placenta	Fatigue, mild dry cough, fever	Not reported	Fetal distress	NA	Alive	Alive	Positive
Liu et al. (15)/10/10	(10) C/S	(10) Positive	(10) Negative	(10) Serologic	(7) Fever, (7) Fatigue, (3) Dyspnea	Not reported	(3) Fetal distress, (1) PROM, (1) Still birth	NA	(10) Alive (One ARDS, intubated, multi organ failure, septic shock, on ECMO)	(9) Alive, (1) stillbirth	(6) Positive, (4) Negative
Li et al. (16)/1/1	C/S	Positive	Negative	Nasopharyngeal, placenta, cord blood, amniotic fluid, neonate urine and feces, breast milk	Dry cough	None	Fetal distress	NA	Alive	Alive	Positive
Khan et al. (17)/3/3	(3) NVD	(3) Positive	(3) Negative	(3) Nasopharyngeal swab	(2) Fever, (3) Cough, (1) chest tightness	(3) Note reported	None	NA	(3) Not reported	(3) Alive	(1) Positive, (2) Negative
Liu et al. (18)/3/3	(2) C/S (1) NVD	(3) Positive	(3) Negative	(3) Nasopharyngeal swab, (1) Placenta, (1) Cord blood, (2) Neonatal urine and feces, (2) Breast milk, (2) Vaginal mucus, (2) Neonate blood	(2) Fever, (2) Cough	(1) Meconium staining, (1) Slightly decreased responsiveness and muscle tone	(1) Fetal distress, (1) Chorioamnionitis	NA	(3) Alive	(3) Alive	(3) Negative
Khan et al. (19)/17/17	(17) C/S	(12) Positive, (5) CT scans in favor of COVID	(15) Negative (2) Positive	(17) Throat swab	(3) Fever, (6) Cough, (3) Diarrhea, (2) Nasal congestion, (2) Shortness of breath, (1) Sputum	(5) Pneumonia	None	NA	(17) Alive	(17) Alive	(3) Positive, (14) Negative
Chen et al. (20)/9/9	(9) C/S	(9) Positive	(6) Negative (3) Not tested	(9) Nasopharyngeal swab, (9) Amniotic fluid, (9) Cord blood, (9) Breast milk sample	(5) Cough, (9) Fever, (2) Malaise, (4) Myalgia, (2) Sore throat	(9) None	(2) Fetal distress (2) PROM	NA	(9) Alive	(9) Alive	(4) Positive, (5) Negative
Chen et al. (21)/4/4	(3) C/S (1) NVD	(4) Positive	(4) Negative	(4) Nasopharyngeal swab	(3) Fever, (2) cough, (2) fatigue, (1) headache, (2) dyspnea	(2) Edema, (2) Rash, (1) Dyspnea	None	NA	(4) Alive	(4) Alive	(4) Negative
Chen et al. (22)/17/17	(17) C/S	(17) Positive	(17) Negative	(17) Nasopharyngeal swab	(4) Fever, (4) Cough, (1) Fatigue, (2) Chest distress, (1) Dyspnea, (1) Diarrhea	None	None	NA	(17) Alive	(17) Alive	(3) Positive, (14) Negative
Dong et al. (23)/1/1	C/S	Positive	Negative	Nasopharyngeal, breast milk	Fever, nasal congestion, dyspnea	None	None	Normal chest CT scan	Alive	Alive	Negative
Xiong et al. (24)/1/1	NVD	Positive	Negative	Nasopharyngeal swab, amniotic fluid, breast milk, rectal swab	Cough, fever, chills	None	None	NA	Alive	Alive	Negative
Wang et al. (25)/1/1	C/S	Positive	Negative	Nasopharyngeal swab, placenta, amniotic fluid, neonatal blood	Fever	None	None	NA	Alive	Alive	Positive
Wang et al. (26)/1/1	C/S	Positive	Positive	Nasopharyngeal swab	Fever	None	None	Thickened lung texture in X-ray, few small pieces of patchy shadows in the upper lobe of right lung in chest CT scan	Alive	Alive	Negative
Zeng et al. (27)/6/6	C/S	Positive	Negative	(6) Nasopharyngeal swab	Mild clinical symptoms	None	None	NA	Alive	Alive	Negative
Zhu et al. (28)/9/10	(7) C/S, (2) NVD	(8) Positive, (1) Negative	(9) Negative, (1) Not tested	(9) Nasopharyngeal swab	(4) Cough, (8) Fever, (1) Sore throat, (1) Diarrhea	(6) Breathing problem, (3) Cyanosis, (2) DIC, (1) Edema, (1) Rash, (2) Feeding intolerance, (1) Bloating, (2) Fever, (1) Vomiting, (2) GI bleeding, (1) Organ failure, (1) Refractory shock	(6) Fetal distress/(3) PROM	(2) NRDS, (2) blurred marking in lungs, (2) patchy GGO in lower lungs, (1) pneumothorax, (3) normal	Alive	(9) Alive, (1) died	(5) Positive, (4) negative
Zamaniyan et al. (29)/1/1	C/S	Positive	Positive	Nasopharyngeal swab (+), cord blood (−), amniotic fluid (+)	Cough, fever, dyspnea, myalgia	Fever	None	NA	Died (ARDS. intubated)	alive	Positive

Diagnosis of the patients was based on a standard protocol that included taking history and physical exam, laboratory findings, and chest CT scans for findings in favor of COVID-19 pneumonia. Out of 83 positive rt-PCRs, 59 samples were taken from the nasopharyngeal area, and 14 were from oropharyngeal. Other studies did not specify the origin of the rt-PCR positive samples.

Laboratory findings revealed 11 leukocytosis (Total reported cases 24), 18 lymphopenia (Total: 28), three thrombocytopenia (Total: 8), 26 elevated C-reactive protein (CRP) and 6 negatives, three elevated aspartate aminotransferase (AST) and 5 elevated alanine aminotransferase (ALT) (Total: 25).

Fifty-nine chest CT scans were in favor of COVI D-19 or viral pneumonia by demonstrating patchy ground-glass opacities in the lungs, and only one was negative, for which RT-PCR was positive for SARS-CoV-2 in the mentioned case. Mothers’ treatment was individualized depending on their condition and the treatment included antibiotic, antiviral, oxygen therapy, and anti-inflammatory therapy. Four patients required mechanical ventilation, one required hemodialysis, and one was on extracorporeal membrane oxygenation (ECMO).

### Outcomes and obstetric characteristics:

Out of 90 pregnant women, 20 were reported to have comorbid disease based on previous medical histories before COVID-19 infection including anemia (N= 5), gestational diabetes mellitus (N=4), hypothyroidism (N=3), preeclampsia (N=3), hypertension (N=2), cholecystitis (N=1), influenza (N=1), thalassemia trait (N=1), and vaginal bleeding in the third trimester (N=1).

The most commonly reported complications were preterm labor (N=29), fetal distress (N=15), pre-mature rupture of membranes (N=6), chorioamnionitis (N=1), and stillbirth (N=1). A study with 17 pregnant cases did not provide any maternal or neonatal complications (19). One patient developed a decrease in O_2_ saturation after delivery, which was resolved with an oxygen mask (9) and two developed post-partum fever (20). Three women were admitted to ICU and required mechanical ventilation and two of them developed acute respiratory distress syndrome (ARDS), and one developed septic shock and multi-organ failure (15). The mentioned patient was on ECMO until the time of the writing of the study. The second mentioned patient who developed ARDS died after delivery due to critical disease (29).

### Mode of delivery:

Infection with COVID-19 was considered as an indication for cesarean section in many cases due to uncertainty of risk of vertical transmission and for relief of abdominal pressure for better respiration (12, 20).

Out of 90 pregnant cases, 81 delivered their baby through cesarean section and 9 through normal vaginal delivery. Although the most common indication for cesarean section was infection with COVID-19, some other indications included fetal distress (N=15), PROM (N=6), history of stillbirth (N=1), stillbirth (N=1), incomplete rotation of the head (N=1), chorioamnionitis and meconium- stained amniotic fluid (N=2) and preeclampsia (N=2).

### Risk of vertical transmission:

Overall, 86 neonates (Five were not tested, and one stillbirth of a total of 92 neonates) were tested for the possibility of vertical transmission. Eighty-two were tested negative in rt-PCR, three were positive, and one was negative at the time of delivery and then tested positive 24 *hr* and 7 days later. Eighty-one samples were taken from oropharyngeal or nasopharyngeal regions, amniotic fluid (N=16), cord blood (N=14) breast milk (N=14), placenta (N=6), neonatal blood (N=4), urine (N=3), feces (N=3), rectal swab (N=2) and mothers vaginal mucus (N=2). In three positive neonates, the only performed test was naso/oropharyngeal swab. One which tested positive after 24 *hr* was also tested positive for the amniotic fluid sample but negative for cord blood at the same time. In four positive neonates, only two were separated from the mother to reduce the risk of infection with COVID-19 (26, 29).

### Neonates outcome:

Based on reviewed studies, out of 92 neo-nates, 1 died (28), and one was born dead (Stillbirth) (15). Moreover, none of these two were tested positive for COVID-19. Neo-nate’s birth weight was ranged from 1520 to 3820 *gr*, eleven had low birth weight (<2500 *gr*), two were small for gesta-tional age (SGA), and one was large for gesta-tional age (LGA). First minute APGAR score was ranged from 7 to 10, and after 5 *min* ranged from 8 to 10. Nineteen neonates reported to have no symptoms and complications. However, breathing problem (N=8), pneumonia (N=5), cyanosis (N=4), fever (N=3), rash (N=3), edema (N=3), DIC (N=2), feeding intolerance (N=2), gastrointes-tinal bleeding (N=2), meconium stain (N=2), decreased responsiveness and muscle tone (N=1), vomiting (N=1), reflux (N=1), bloat-ing (N=1), organ failure (N=1) and refractory shock (N=1) were reported in others. None of the neonates received antiviral treatment. Chest x-ray was performed in 10 symptomatic patients among which seven revealed abnor-mal findings such as Neonatal Respiratory Distress Syndrome (NRDS) (N=2), patchy shadows (N=2), blurred marking (N=2) and thickening of lung texture (N=1) in both lungs. Furthermore, one chest CT scan was done in a positive neonate without symptoms for COVID-19, which showed few small pieces of patchy shadows in the upper lobe of the right lung (26). Out of 4 positive cases, one had no clinical symptoms (26), and one presented with fever (29). The other two were only reported to be suspected for COVID-19 with no further explanations (19). Further-more, 5 neonates who reported to have pneu-monia were tested negative for COVID-19 (19).

## Discussion

The study results revealed that the most common symptoms among pregnant women were fever, cough, and dyspnea, similar to non-pregnant patients (30). The vast majority of women delivered their baby through cesarean section and they were discharged from hospital with no significant or minor complications such as mild preterm or fetal distress. Besides, our analysis revealed that neonates with positive COVID-19 infection were diagnosed mostly by naso/oropharyngeal swab test. Moreover, most of the neonates presented with breathing problems followed by pneumonia, and only two of them died, but none of these two was tested positive for COVID-19. It seems that the possibility of vertical transmission in pregnant women with COVID-19 infection is lower than expected.

The first case of infection with COVID-19 was recognized in November 2019 based on Chinese data, and World Health Organization (WHO) has provided a day-to-day timeline report of the development of the current coronavirus pandemic (31, 32). Numerous studies have been performed in order to describe the epidemiology, characteristics, and prevention of COVID-19 infection (1, 33).

Zhang et al. (34) revealed that droplets, contact, aerosol, and fecal–oral transmissions are the main transmission routes in COVID-19 infection. On the other hand, based on the retrospective study by Chen et al., (20) the amniotic fluid, cord blood, neonatal throat swab, and breastmilk samples from newborn neonates of COVID-19 positive mothers were negative for SARS-CoV-2 and there are few review studies about the possibility of the vertical transmission in pregnancy (31, 35, 36). On the other hand, based on Chinese reports, chest CT scan had higher sensitivity compared to RT-PCR, with a mean interval time between the initial negative to positive RT-PCR results of 5.1± 1.5 days, although, CT use must be evaluated by the physician’s knowledge especially for pregnant women and children to prevent patient consequent exposure (37).

The physiologic and immunologic changes during the pregnancy may increase the risk of neonatal defects from many pulmonary viral infections (38). In recent COVID-19 studies, it was revealed that most infected pregnant women, were from Wuhan, China and/or had close contact with pa-tients with lab-confirmed COVID-19 infection (38–40). However, in a previous study, definite practices such as avoidance of aerosol-generating procedures in virus-infected patients could be offered as an option to all pregnant women by the Centers for Disease Control and Prevention (CDC); CDC stated that pregnant women should rigorously follow the same measures that were suggested for health care providers (41). Moreover, the creation of appropriate self-care and hygiene practice and weight gain near the end of pregnancy are the important ways to reduce the morbidity and complication of the COVID-19 infection in pregnant women (42).

In the previous study by Lam et al., (43) with experience of infections caused by similar COVID-19 pathogens, the clinical course and results of pregnancy in women with SARS were assessed. It was revealed that although pregnancy seemed to have no influence on clinical symptoms after symptom onset, complications including sepsis, acute kidney injury, and disseminated intravascular coagulation (DIC) were considerably increased in pregnant women. Besides, they observed that the usage rate of mechanical ventilation and mortality were more common among them than others. In line with the result of this study, Mullins et al. (44) reported that in COVID-19 pregnant women, the maternal complaint is not as severe, and the concerns will be based more on individual symptoms. However, in the patients with SARS and MERS infections, they reported that delivery was most often indicated by maternal hypoxemia.

Lu et al. (45) reviewed three neonates and 230 children in their study with COVID-19 positive mothers and reported that most of the children had mild disease conditions and all neonates were healthy; finally, they concluded that SARS- CoV-2 could not be transplacen-tally transmitted from mother to the newborn. Karimi-Zarchi et al. (46) reviewed 31 pregnant women with COVID-19 positive tests and showed that there is no evidence for intrauterine transmission of this infection from infected mothers to their fetuses but, infected pregnant women may be at increased risk of pulmonary complications. Another review study by Panahi et al. reported that vertical transmission in pregnancy is rare, and some symptoms of COVID-19 in pregnant mothers are not different from those of non-pregnant (47). Moreover, in a systematic review by Zaigham and Andersson, after evaluation of 108 pregnancies in the COVID-19 pandemic period, they revealed that most of the women had fever, coughing, and lymphocyto-penia with elevated C-reactive protein and also, most of them delivered by cesarean section; their results are in line with the results of our study (48).

However, similar to some recent studies, the vast majority of COVID-19 positive pregnant women recovered, and their neonate’s tests for SARS-CoV RNA were negative with no evidence of vertical transmission (46, 49). Four COVID-19 positive neonates were reported in this study by naso/oropharyngeal swab test (19, 26, 29). One had no clinical symptoms (26), and one presented with fever (29). The other two were only reported to be suspected for COVID-19 with no further explanations (19) In addition, in all these cases, the possibility of close contact history cannot be excluded, although, no consistent document is as yet presented to reveal the possibility of vertical transmission of COVID-19 infection from the mothers to the neonates.

Hantoushzadeh et al. (50) reported that among nine pregnant women in their second or 3rd-trimester stages with positive SARS-CoV-2, seven patients died, one remained critically ill and ventilator-dependent, and one recovered after long-term hospitalization. Their results are significantly different from the results of this study, and it means that long-term follow-up may be necessary for the evaluation of maternal consequences.

### Limitations:

The current study had certain limitations. First, all of the available studies were in forms of case series, case reports, or letters containing a case description; and there were not a cohort study or retrospective studies in this regard. Second, most of the available studies were from China and had population biases. Third, included studies had heterogeneity, and some outcomes were missed in some of the studies.

## Conclusion

In our review of the recent literature, the clinical outcome was mostly satisfactory for both mothers and neonates. There was not a significant difference in the clinical characteristics of pregnant women and non-pregnant COVID-19 patients. COVID-19 infection has caused higher incidence of fetal distress and premature labor in pregnant women. Besides, the naso/oropharyngeal swab test is a rapid diagnostic test in the detection of COVID-19 infection in neonates. Although the possibility of vertical transmission in pregnant women with COVID-19 infection is rare, four neonates’ test results for COVID-19 infection were positive in this review.

## Supplementary Appendix

The keywords detail and full search strategy used in PubMed are as follows: Both medical subject headings (MeSH) and keywords: “2019 novel coronavirus infection” OR “COVID-19” OR “COVID19” OR “coronavirus disease 2019” OR “nCoV infection” OR “2019-nCoV” OR “2019 novel coronavirus” OR “2019 coronavirus” OR “novel coronavirus” OR (2019 AND coronavirus) AND (“vertical transmission” OR trimester OR delivery OR cesarean OR gestational OR pregnant OR labor OR newborn OR maternal OR perinatal OR neonatal OR pregnancy OR “vaginal delivery” OR neonate OR mother OR babies OR baby OR breastfed OR breastfeed OR intrauterine OR born OR fetal OR “cord blood” OR placenta OR placentas OR peripartum OR obstetricians OR gynaecologists OR “preterm birth” OR preterm OR fetus OR “C-section” OR “C section” OR “C-sections” OR “postcesarean section” OR childbirth OR “mother-to-child transmission” OR pregnancies OR gestation OR placentome OR placentomes OR placentoma OR (transmission AND vertical) OR (transmission AND fetomaternal) OR (birth AND premature) OR “infectious disease transmission, vertical” OR “pregnancy trimesters” OR “pregnancy trimester, third” OR “delivery, obstetric” OR “cesarean section” OR “labor, obstetric” OR “infant, newborn” OR pregnancy OR fetus OR “fetal blood” OR placenta OR “premature birth”.
